# Developing and Pilot Testing a Spanish Translation of CollaboRATE for Use in the United States

**DOI:** 10.1371/journal.pone.0168538

**Published:** 2016-12-21

**Authors:** Rachel C. Forcino, Nitzy Bustamante, Rachel Thompson, Sanja Percac-Lima, Glyn Elwyn, Diana Pérez-Arechaederra, Paul J. Barr

**Affiliations:** 1 The Dartmouth Institute for Health Policy & Clinical Practice, Lebanon, NH, United States of America; 2 Harvard Medical School, Boston, MA, United States of America; 3 Massachusetts General Hospital Chelsea HealthCare Center, Chelsea, MA, United States of America; 4 Primary Care Research Unit, The Alamedilla Health Center. Biomedical Research Institute of Salamanca (IBSAL), Salamanca, Spain; 5 Centro Universitario San Rafael—Nebrija, Madrid, Spain; University of California San Diego, UNITED STATES

## Abstract

**Background/Aim:**

Given the need for access to patient-facing materials in multiple languages, this study aimed to develop and pilot test an accurate and understandable translation of CollaboRATE, a three-item patient-reported measure of shared decision-making, for Spanish-speaking patients in the United States (US).

**Method:**

We followed the Translate, Review, Adjudicate, Pre-test, Document (TRAPD) survey translation protocol. Cognitive interviews were conducted with Spanish-speaking adults within an urban Massachusetts internal medicine clinic. For the pilot test, all patients with weekday appointments between May 1 and May 29, 2015 were invited to complete CollaboRATE in either English or Spanish upon exit. We calculated the proportion of respondents giving the best score possible on CollaboRATE and compared scores across key patient subgroups.

**Results:**

Four rounds of cognitive interviews with 26 people were completed between January and April 2015. Extensive, iterative refinements to survey items between interview rounds led to final items that were generally understood by participants with diverse educational backgrounds. Pilot data collection achieved an overall response rate of 73 percent, with 606 (49%) patients completing Spanish CollaboRATE questionnaires and 624 (51%) patients completing English CollaboRATE questionnaires. The proportion of respondents giving the best score possible on CollaboRATE was the same (86%) for both the English and Spanish versions of the instrument.

**Discussion:**

Our translation method, guided by emerging best practices in survey and health measurement translation, encompassed multiple levels of review. By conducting four rounds of cognitive interviews with iterative item refinement between each round, we arrived at a Spanish language version of CollaboRATE that was understandable to a majority of cognitive interview participants and was completed by more than 600 pilot questionnaire respondents.

## Introduction

Making efforts to engage and activate patients without addressing diverse patient needs with regard to racial, ethnic, and linguistic differences can exacerbate existing health disparities[1–4]. It is therefore important to ensure that tools and measures are available in a range of languages. CollaboRATE is a three-item patient-reported measure of shared decision-making within clinical encounters[5,6]. As 13 percent of United States (US) residents speak Spanish at home[7], a Spanish version of CollaboRATE can better serve this segment of the US population and enhance representativeness of respondent samples to better fit US demographics. This is of particular importance as language, particularly limited English proficiency, is a well-recognized barrier to accessing and receiving high-quality health care in the US[8]. For example, Morales[9] and Baker[10] found poorer patient-provider communication among Spanish-speaking patients than among their English-speaking counterparts. However, others have found no difference in satisfaction with patient-provider communication across languages[11–13]. Differences in measurement instruments used may contribute to these mixed findings.

Given the importance of rigorous translation and evaluation procedures paired with a need for access to patient resources in multiple languages, this study aimed to develop and pilot test a Spanish translation of CollaboRATE for use by Spanish-speaking patients in the US.

## Materials and Methods

This project consisted of three phases: 1) translation and review of the English version of CollaboRATE by professional bilingual translators, 2) refinement of a Spanish language version of CollaboRATE through interviews with end users, and 3) pilot testing of the Spanish language questionnaire with adult patients in an internal medicine clinic.

### Team translation

Questionnaires fielded in multiple languages are susceptible to measurement bias resulting from inconsistencies between questionnaire versions[14–16]. Translation accuracy is essential to ensuring that differences observed between linguistic groups are actual differences rather than the result of measurement bias. Therefore, best practices in cross-cultural survey research require sound translation procedures followed by thorough pre-testing, evaluation, and refinement of the translated material[17,18].

While back-translation as recommended by Brislin[19] has been commonly relied upon in health services and survey translation projects[14,20], there is movement toward team and committee-based translation procedures in an effort to avoid literal translation and emphasize cultural appropriateness in the target language[18,21,22]. Guidelines for questionnaire translation, such as the Translate, Review, Adjucate, Pre-test, Document (TRAPD) protocol, recommend a team translation process consisting of multiple independent translations, subsequent review by a third individual, and finally an adjudicator’s nomination of a version of the questionnaire for cognitive interviews and pilot testing with the target population[18,23].

In keeping with the TRAPD protocol, we engaged two professional translators to independently draft Latin American Spanish translations of the CollaboRATE questions and 10-point response scale intended for Spanish-speaking individuals in the United States. A third independent translator from the same vendor reviewed those translations and incorporated favored components of the initial drafts into a third version. A bilingual member of the research team with subject matter expertise in shared decision-making (DP) reviewed the third draft and, after making refinements, established a final version for cognitive interview pre-testing.

### Cognitive interviews

#### Procedure

Cognitive interviews play an important role in evaluating survey instruments, particularly when translation is involved[17,24]. Cognitive interviews involve in-depth consultation with members of the target audience to explore comprehension, interpretability, and cultural appropriateness of questionnaire translations[17]. We conducted multiple rounds of structured cognitive interviews with iterative item refinement between each round.

The Spanish interview guide (S1 File) was adapted from the original CollaboRATE cognitive interview guide[6] by a bilingual member of the research team (NB) and included questions like ‘Is the question clear?’, ‘In your own words, what do you think the question is asking?’, and ‘What does the following phrase mean to you?’. All interview participants received printed copies of the CollaboRATE items being discussed; where participants preferred to hear the items spoken aloud, they were read twice by a native Spanish speaking interviewer before proceeding to the cognitive interview questions. Demographic information including age, gender, occupation, and level of education was also collected. A native US speaker of both Spanish and English (NB) conducted and analyzed the interviews. With participant permission, interviews were audio-recorded to facilitate analysis.

#### Site

Interviews were conducted at the adult internal medicine clinic of an urban Massachusetts community health center.

#### Participants and recruitment

A native speaker of both English and Spanish approached individuals in the waiting room and staff areas of the health center’s adult internal medicine clinic inviting them to participate in the study. All Spanish-speaking adults (18 years or older) within the clinic were eligible to participate. Participants reviewed a Spanish-language information sheet and provided verbal consent. Participants were compensated with $10 grocery store gift cards.

#### Interview analysis

Analysis focused on participants’ understanding and interpretation of each item. As detailed by Levin[17], we used a two-step analytic approach that first developed item-level interview summaries of the comments made and then grouped problems into categories to facilitate item revision. Following each round of interviews, NB suggested refinements based upon participants’ feedback. These changes were discussed among the research team, with consensus dictating whether to retain or discard each modification. Refined items were then tested in a new round of interviews. Interview rounds proceeded until a majority of participants expressed an understanding of the items that matched their intended meaning.

### Pilot data collection

#### Site

Pilot data collection took place within the same adult internal medicine clinic where cognitive interviews were conducted.

#### Recruitment

Adult patients with weekday visits at the internal medicine clinic between May 1 and May 29, 2015 were eligible to participate. Patients with visits on Saturdays were excluded as the clinic is minimally staffed for only urgent appointments on weekends.

Following visits, bilingual medical assistants (MAs) provided each patient with a one-page survey (S2 File) along with other routinely provided paperwork. Separate surveys were available in English and Spanish. The MA determined which version of the survey to give to each patient based on pre-visit communication with the patient. The survey included the three CollaboRATE items in addition to supplementary questions asking the patient’s age, gender, and whether an interpreter was present in the visit. MAs marked the appropriate clinician’s initials on each survey and asked patients to complete the survey and deposit it in a secure box at the MA station. In addition, reception staff prompted patients to ensure they had deposited the survey before leaving the clinic. To maximize adherence to data collection protocols, one-time payments of $100 were awarded to each MA at the end of the one-month study period.

#### Data analysis

We assessed the proportion of respondents across key subgroups including gender, age, questionnaire language, and interpreter use. We also examined these demographics by questionnaire language. The CollaboRATE score represents the proportion of respondents giving the best score possible on CollaboRATE. This scoring technique is a strategy for aiding interpretation notwithstanding potential ceiling effects common in patient-reported measurement of clinician performance[5,25]. CollaboRATE scores were calculated for all responses as well as for each questionnaire language subgroup.

### Ethical approvals

Dartmouth College’s Committee for the Protection of Human Subjects reviewed and approved this study and its consent procedures. Partners Healthcare Institutional Review Board reviewed this study and considered it exempt from further review. To minimize the identifiable participant information collected in this minimal risk research study, written consent requirements were waived. Prospective interview participants were given an information sheet (S1 File) and asked to provide verbal consent prior to participation. Verbal consent was documented via audio recording.

## Results

### Translation

Table 1 outlines the evolution of items throughout the translation process. The two independent translations maintained consistency in general sentence structure, though they varied in choice of vocabulary.

**Table 1:** Item progression: Translation process.

	Original English items	Independent translation 1	Independent translation 2	Reviewer's version	Adjucator's version
**Item 1**	How much effort was made to help you understand your health issues?	¿Cuánto esfuerzo se realizó para ayudarlo a comprender sus problemas de salud?	¿Cuánto esfuerzo se hizo para ayudarle a entender sus problemas de salud?	¿Cuánto esfuerzo se hizo para ayudarle a entender sus problemas de salud?	a. ¿Cuánto esfuerzo se hizo para ayudarle a entender sus problemas de salud?
					b. ¿Cuánto esfuerzo se hizo para ayudarle a entender su estado de salud?
**Item 2**	How much effort was made to listen to the things that matter most to you about your health issues?	¿Cuánto esfuerzo se realizó para escuchar las cosas que le importan más a usted acerca de sus problemas de salud?	¿Cuánto esfuerzo se hizo para escucharle sobre las cosas de más importancia para usted sobre sus problemas de salud?	¿Cuánto esfuerzo se hizo para escucharle sobre las cosas de mayor importancia para usted sobre sus problemas de salud?	a. ¿Cuánto esfuerzo se hizo para escuchar las cosas que más le importan (a usted) sobre sus problemas de salud?
					b. ¿Cuánto esfuerzo se hizo para escuchar las cosas que más le importan (a usted) sobre su estado de salud?
**Item 3**	How much effort was made to include what matters most to you in choosing what to do next?	¿Cuánto esfuerzo se realizó para incluir lo que es más importante para usted en la decisión de qué hacer a continuación?	¿Cuánto esfuerzo se hizo para incluir lo que es más importante para usted en la elección del próximo paso a tomar?	¿Cuánto esfuerzo se hizo para incluir lo de mayor importancia para usted en la elección del próximo paso a tomar?	¿Cuánto esfuerzo se hizo para incluir lo que a usted le importa más en la elección qué hacer a continuación?
**Response scale**	No effort was made—Every effort was made	Ningún esfuerzo—Se realizaron todos los esfuerzos	Ningún esfuerzo—Se hizo todo el esfuerzo posible	Ningún esfuerzo—Se hizo todo el esfuerzo posible	a. Ningún esfuerzo—Todo esfuerzo
					b. No se hizo ningún esfuerzo—Se hizo todo el esfuerzo posible

The reviewer created a third version of the questionnaire by incorporating elements of each previous version and making modifications as appropriate. While item 1 was wholly adopted from independent translation 2, the reviewer adapted items 2 and 3 from independent translation 2 to refer to ‘mayor importancia’ rather than ‘más importancia’ when asking about the ‘things that matter most’.

In the adjudication stage, modifications to the response scale labels were recommended to maintain consistency in structure and vocabulary between the two extremes of the scale. Additional changes at this stage included a transition in the third item from ‘lo de mayor importancia para usted’ to ‘lo que a usted le importa más’ to describe that which is most important to the respondent.

### Cognitive interviews

#### Participants

Participants’ demographic characteristics are presented in Table 2. All participants were Hispanic and native Spanish speakers. Most participants were women (20/26, 77%) and nearly half (12/26, 46%) had educational attainment less than a high school diploma. All participants who provided their age (22/26, 86%) were under 65, with approximately half of those individuals under age 45 (10/22, 45%).

**Table 2:** Participant characteristics.

	Round 1	Round 2	Round 3	Round 4
	(n = 8)	(n = 6)	(n = 6)	(n = 6)
**Gender**				
Male	3	1	0	1
Female	4	5	6	5
Did not indicate	1	0	0	0
**Age**				
18–44	4	0	2	4
45–64	2	5	3	2
65+	0	0	0	0
Did not indicate	2	1	1	0
**Education**				
Less than high school	2	1	5	4
High school diploma	2	0	0	0
Some college	1	1	1	0
Bachelors degree	0	1	0	1
Postgraduate/Professional degree	1	1	0	0
Did not indicate	2	2	0	1
**Employment status**				
Paid employment	5	3	2	3
No paid employment	1	2	3	3
Did not indicate	2	1	1	0
**Ethnicity**				
Hispanic	2	2	4	3
Latino/Latina	1	1	0	2
Other	3	2	2	1
Did not indicate	2	0	0	0

#### Interviews

Four rounds of cognitive interview were completed between January and April 2015. After three rounds of interviews, we reached consensus on items 1 and 2. One additional round of interviews was required for item 3. Table 3 presents versions of each item as they were tested in each round of cognitive interviews.

**Table 3:** Item progression: Cognitive interviews.

	Original English items	Round 1	Round 2	Round 3	Round 4	Final version for pilot testing
**Item 1**	How much effort was made to help you understand your health issues?	a. ¿Cuánto esfuerzo se hizo para ayudarle a entender sus problemas de salud?	a. ¿Cuánto se trató de ayudarle a entender sus problemas de salud?	a. ¿Cuánto se trató de ayudarle a entender sus problemas de salud?	N/A	¿Cuánto cree que se hizo para ayudarle a entender sus problemas de salud?
		b. ¿Cuánto esfuerzo se hizo para ayudarle a entender su estado de salud?	b. ¿Cuánto se trató de ayudarle a entender su estado de salud?	b. ¿Cuánto cree que se hizo para ayudarle a entender sus problemas de salud?		
**Item 2**	How much effort was made to listen to the things that matter most to you about your health issues?	a. ¿Cuánto esfuerzo se hizo para escuchar las cosas que más le importan (a usted) sobre sus problemas de salud?	a. ¿Cuánto se trató de escucharle cuando usted comunico las cosas que mas le importan sobre sus problemas de salud?	a. ¿Cuánto se trató de escucharle cuando usted comunicó las cosas que más le importan sobre sus problemas de salud?	N/A	¿Cuánto cree que se hizo para escucharle cuando usted comunicó lo que más le importa acerca de sus problemas de salud?
		b. ¿Cuánto esfuerzo se hizo para escuchar las cosas que más le importan (a usted) sobre su estado de salud?	b. Cuando usted comunicó las cosas que mas le importan sobre sus problemas de salud, ¿cuánto se trató de escucharle?	b. ¿Cuánto cree que se hizo para escucharle cuando usted comunicó lo que más le importa acerca de sus problemas de salud?		
**Item 3**	How much effort was made to include what matters most to you in choosing what to do next?	¿Cuánto esfuerzo se hizo para incluir lo que a usted le importa más en la elección qué hacer a continuación?	¿Cuánto se trató de incluir lo que más le importa a usted al escoger cómo seguir adelante?	a. ¿Cuánto se trató de incluir lo que más le importa a usted al escoger como seguir adelante?	¿Del 1 al 9, cuánto cree que se hizo para incluir lo que más le importa a usted cuando se escogió el siguiente paso?	¿Cuánto cree que se hizo para incluir lo que más le importa a usted cuando se escogió el siguiente paso?
				b. ¿Cuánto cree que se hizo para tomar en cuenta lo que más le importa al escoger como seguir adelante?		
**Minimal response**	No effort was made	a. Ningún esfuerzo	a. Todas igual	a. No se hizo en absoluto	N/A	No se hizo
		b. Ningún esfuerzo para nada	b. Nada	b. No se hizo para nada		
		c. No se hizo ningún esfuerzo	c. No se trató para nada	c. No se hizo		
		d. Ningun	d. No se trató	d. Nada		
**Maximal response**	Every effort was made	a. Todo esfuerzo	a. Se trató lo mejor posible	a. Se hizo lo mejor posible	N/A	Se hizo lo mejor posible
		b. Un gran esfuerzo	b. Se trató lo major que se pudo	b. Se hizo lo mejor que se pudo		
		c. Si eso todo esfuerzo		c. Se hizo bastante		
		d. Un gigante esfuerzo		d. Bastante		

In round 1 of interviews, item 1 was understood as intended by four of the eight participants. Of those who did not understand the question, two cited the term ‘esfuerzo’ (effort) as problematic. As such, ‘esfuerzo’ was replaced with ‘trató’ (tried) for interview round 2. Three out of eight participants favored the phrase ‘problemas de salud’ (health problems) in this round, while five preferred ‘estado de salud’ (state of health). Accordingly, both phrases were tested again in round 2.

In round 2, the refined version of item 1 was understood as intended by only one participant (1/6 participants). Quasi-homophones, or words which are pronounced similarly but confer different meanings, impeded comprehension for two participants (2/6 participants), where ‘cuándo’ was mistaken for ‘cuánto’ and ‘tardo’ for ‘trato’. Thus, alternative wording to ‘cuánto se trató’ was presented in round 3. Two additional participants cited confusion about the grammatical subject of the question (2/6 participants) as the translation retains ambiguity surrounding whose effort each item is intended to assess. Three participants preferred the phrase ‘problemas de salud’ over ‘estado de salud’ (3/6 participants), while three cited no preference (3/6 participants). Despite an overall lack of clarity among most participants, the phrase ‘ayudarle a entender’ was well understood by participants to mean ‘help you understand’.

Five of the six round 3 participants found item 1b to be clear and understandable. Item 1a posed problems in that some participants understood ‘trato’ to mean ‘treatment’ rather than ‘trying’; therefore item 1b was adopted as the final version for pilot testing.

Item 2 was well understood by five of eight participants in round 1, though two participants again cited ‘esfuerzo’ as a problematic term. There was also confusion on whether it was the patient or the provider who was expected to ‘escuchar a las cosas que más le importan a usted’ (listen to the things that matter most to you). As the original English version of the questionnaire included intended ambiguity around the subject of each CollaboRATE question[6], this finding did not spur changes in round 2.

In round 2, while item 2 was well understood by three participants (3/6 participants), two expressed concern with the length of the question. The phrase ‘las cosas que más le importan sobre su estado/sus problemas de salud’ in particular was considered too long. Accordingly, round 3 adopted the shorter phrase ‘lo que más le importa’ to replace ‘las cosas que más le importan’.

All round 3 participants found item 2 clear and understandable (6/6 participants). As both 2a and 2b had equally acceptable comprehension, 2b was adopted as the final version for pilot testing to maintain consistency with item 1.

In round 1, item 3 was well understood by only one participant (1/8 participants). Three cited the phrase ‘elección que hacer a continuación’ (choosing what to do next) as unclear, attributing the lack of clarity to both sentence structure and improper use of the word ‘elección’ (choice). Round 2 therefore replaced ‘elección’ with ‘al escoger’.

Item 3 was again poorly understood in round 2, with five participants finding the item confusing (5/6 participants) despite three participants understanding that it refers to a situation in the future (3/6 participants). While the term ‘incluir’ contributed to the confusion, ‘al escoger cómo seguir adelante’ was well understood. Additionally, the verb structure ‘se hizo’ was reintroduced in round 3 based on a participant’s suggestion.

Round 3 saw improved comprehension, though three participants (3/6 participants) misinterpreted the phrase ‘seguir adelante’ to refer to an ‘effort’ or ‘fight’ rather than the intended ‘next steps’. As such, item 3 was rephrased to refer instead to ‘el siguiente paso’ and tested in a fourth round of interviews.

In round 4, a majority of respondents reported item 3 to be clear and understandable (4/6 participants). Despite some confusion with the phrase ‘cuando se escogió el siguiente paso’, three of the six participants expressed an understanding of the phrase that was consistent with our intent. Item 3 was therefore adopted at this stage for pilot testing.

Of the four response scale options presented, participants in round 1 favored ‘ningún’ (none) as the lower anchor by a slight margin (3/8 participants), and half (4/8 participants) preferred ‘un gran esfuerzo’ (a large effort) as the upper anchor. As use of the term ‘esfuerzo’ was considered problematic elsewhere in the questionnaire, round 2 adopted the term ‘se trató’ in place of ‘esfuerzo’.

In round 2, participants favored ‘no se trató’ (2/6 participants) as the lower anchor and ‘se trató lo mejor que se pudo’ (3/6 participants) as the corresponding upper anchor. As round 3 introduced ‘se hizo’ as an alternate phrasing in items 1 through 3, this new language was also tested as part of the response scale in round 3.

Participants in round 3 favored ‘no se hizo’ (2/6 participants) as the lower anchor and equally preferred ‘bastante’ (2/6 participants) and ‘se hizo lo mejor que se pudo’ (2/6 participants) as upper anchors. To maintain structural consistency with the lower anchor, ‘se hizo’ was maintained in the final version of the response scale. However, to simplify syntax, the phrase ‘se hizo lo mejor posible’ was adopted as the final version for pilot testing.

#### Pilot data

Of the 1687 eligible patients during the study period, 1230 (73%) completed the survey. 606 patients (49%) completed the Spanish survey, while the remaining 624 (51%) completed the English survey. A large proportion of eligible patients with Spanish documented in clinic records as their primary language completed a Spanish questionnaire (606/760, 80% response rate). Table 4 presents a demographic profile of respondents by questionnaire language. Among Spanish-speaking patients with appointments during the study period, 15 nationalities were represented–though we lack data on the specific nationality of each respondent.

**Table 4:** Pilot data collection: respondent profile.

Characteristic	Total % (N)	English questionnaire users % (N)	Spanish questionnaire users % (N)
**Gender**	(1223)	(623)	(600)
Men	31% (375)	37% (231)	24% (144)
Women	69% (848)	63% (392)	76% (456)
**Age**	(1223)	(623)	(600)
Under 18	1% (7)	0% (0)	1% (7)
18–24	8% (97)	10% (65)	5% (32)
25–34	16% (200)	15% (96)	17% (104)
35–44	16% (198)	12% (73)	21% (125)
45–54	21% (251)	18% (110)	24% (141)
55–64	16% (198)	19% (121)	13% (77)
65–74	12% (149)	14% (86)	11% (63)
75–84	7% (88)	8% (50)	6% (38)
85+	3% (35)	4% (22)	2% (13)
**Questionnaire language**	(1230)	51% (624)	49% (606)
**Interpreter use**	(1142)	(586)	(556)
Yes	11% (131)	12% (69)	11% (62)
No	89% (1011)	88% (517)	89% (494)

CollaboRATE scores did not vary between questionnaire languages, with 86% of respondents to both the English and Spanish questionnaires providing highest-possible scores (English 95% CI 84–89%; Spanish 95% CI 83–89%; *χ*^2^ = 0.015, p = 0.901).

## Discussion

### Key findings

Despite the thorough translation process adopted prior to pre-testing the Spanish version of CollaboRATE with the intended respondent population, the subsequent cognitive interviews revealed significant variation in comprehension. By conducting four rounds of cognitive interviews with iterative item refinement between each round, we arrived at a Spanish language version of CollaboRATE that was understandable to a majority of cognitive interview participants and was completed by more than 600 pilot questionnaire respondents.

### Strengths and limitations

This study’s methodological strengths lie in its approach to translation and questionnaire pre-testing via multiple iterative rounds of cognitive interviews with the target audience, and in the diversity of the sample. The single study site may limit generalizability of the findings and, therefore, the appropriateness of the translation for alternate sites and regions within the US. The interviewer was most familiar with Peruvian Spanish, though she relied on input from participants of various ethnic and linguistic backgrounds in proposing item revisions. Nevertheless, the issue of generalizability is exacerbated by the linguistic and cultural diversity of the US Spanish-speaking population. While additional research in other areas of the country would confirm the appropriateness of this translation among the diverse Spanish-speaking US population, previous studies have similarly focused on attaining diverse representation of Spanish speakers within a single US location[26].

Additionally, we lack data on language concordance between the patients and clinicians who participated in the pilot data collection phase of the study. As limited English proficiency has been shown to relate to suboptimal SDM in some clinical settings[27] and only 43 percent of patients attending the clinic during the pilot study period report English as their primary language, this leaves a potential for artificially lower English language questionnaire scores among non-English speaking patients completing English-language questionnaires. Given the pilot nature of this study without full information on language concordance between patients and clinicians, further research is needed to address whether a lack of language concordance among English-language respondents and their clinicians impacts our conclusions.

### Context and implications

#### Acceptability

The high response rate among Spanish speakers to the Spanish-language questionnaire observed during the pilot period (80%) provides preliminary evidence of acceptability for routine CollaboRATE administration among Spanish-speaking patients. Additionally, as only one respondent to the Spanish-language questionnaire submitted an incomplete CollaboRATE response, the high rate of completion suggests that the Spanish translation is easy for this population to complete.

### Conclusion

High response rates for the Spanish-language CollaboRATE survey support its acceptability as a patient-reported measure of shared decision-making. However, more research is needed to assess the generalizability of this translation to other US Spanish-speaking populations and confirm the psychometric properties of the Spanish translation. Further testing of this version is currently underway in a large multi-center trial. Further iterations of this translation are available at www.collaboratescore.org.

## Supporting Information

S1 File Interview guide. (PDF)

S2 File Pilot survey form. (PDF)

S1 Table Data sharing: CollaboRATE question 1 pilot data tabulation. (PDF)

S2 Table Data sharing: CollaboRATE question 2 pilot data tabulation. (PDF)

S3 Table Data sharing: CollaboRATE question 3 pilot data tabulation. (PDF)

S4 Table Data sharing: Respondent age pilot data tabulation. (PDF)
