# Supplementary material for: Developing and Pilot Testing a Spanish Translation of CollaboRATE for Use in the United States
Source: PLoS One. 2016 Dec 21;11(12):e0168538. doi: 10.1371/journal.pone.0168538 (PMC5176178; doi:10.1371/journal.pone.0168538)
Supplement: S1 File — (PDF) [file pone.0168538.s001.pdf]

Hoja de Información

Estudio de entrevista cognitiva de CollaboRATE en español. Una medida traducida del enfoque orientado al paciente durante encuentros clínicos.

Este es un proyecto de investigación. Estamos tratando de crear tres preguntas para hacerle a las personas sobre su experiencia con su proveedor médico o doctor. **Nosotros entendemos que tal vez su médico no labore en MGH Chelsea**, pero nos gustaría que nos diga lo que usted piensa de las preguntas en nuestra encuesta.

¿Qué va a pasar durante la entrevista?

Le pedirémos que lea un párrafo. Cuando termine de leerlo, nos gustaría que lea unas preguntas. No le estamos pidiendo que usted conteste las preguntas, sino que nos ayude a hacerlo mejor. Le vamos a preguntar sobre las preguntas que usted acabó de leer. Voy a preguntar cosas tales como:

- ¿Qué entiende usted por esta pregunta?
- ¿Tienen sentido las palabras usadas en la pregunta?
- ¿Hay algo en la pregunta que usted encuentre confuso? Cómo preguntaría usted esta pregunta?

Nuestra meta es determinar si las preguntas son claras y faciles de comprender, así que por favor no dude en decirnos cuando algo no quede claro. Sería una gran ayuda. Por favor tome en cuenta que nosotros realmente queremos escuchar todas sus opiniones y reacciones.

Haremos esto por aproximadamente 10-20 minutos, a menos que ya no tengamos nada que preguntarle o si usted prefiere no contestar más preguntas. Cuando terminemos, le daremos un certificado de valor de $10 a Market Basket.

Usted puede decidir no contestar algunas o ninguas de las preguntas. Con su permiso, vamos a grabar la entrevista. Usted puede pedir que pare la grabación en cualquier momento. Las grabaciones serán transcritas y guardadas de forma segura en el Dartmouth Center for Health Care Delivery Science. No vamos a obtener ninguna información que le pueda identificar.

Este proyecto no le va a benificiar, y como no vamos a obtener información que le pueda identificar, no hay ningún riesgo en cuanto a su participación. **Si usted es paciente aquí, esta información no será parte de su cuidado médico y no será usada en su cuidado médico ni formará parte de su historial médico. Ayudarnos a crear estas preguntas es solamente para investigación.**

Si usted tiene alguna pregunta sobre este proyecto, puede comunicarse con la investigadora principal, la doctora Sanja Percac-Lima. Su teléfono es 617-889-8580.

Interview Guide

Date ________ Interview#_____________ Interviewer Initials___________

Para empezar, queremos que usted se imagine que usted es el siguiente paciente:

"Usted ha ido a una clínica por un problema de salud para ver que se puede hacer. Usted sabe que hay unos cuantos tratamientos diferentes disponibles pero usted quiere saber más acerca de ellos. A su salida, se le pide que complete este cuestionario. El cuestionario podria venir en una página de papel, o en una pantalla táctil o en su teléfono. A usted no se le pide dar su nombre."

Recuerde, por favor no trate de contestar las preguntas. Nosotros queremos averiguar cuan claras y entendibles son las preguntas.

Aquí están algunas preguntas que se le podrían preguntar:

1. ¿Cuánto esfuerzo se hizo para ayudarle a entender (sus problemas de salud)/(su estado de salud)?

2. ¿Cuánto esfuerzo se hizo para escuchar las cosas que más le importan (a usted) sobre (su estado de salud)/(sus problemas de salud)?

3. ¿Cuánto esfuerzo se hizo para incluir lo que a usted le importa más en la elección qué hacer a continuación?

Por favor lea las cuatro escalas de respuestas abajo.

1	2	3	4	5
Ningún esfuerzo				Todo esfuerzo

1	2	3	4	5
Ningún esfuerzo para nada				Un gran esfuerzo

1	2	3	4	5
No se hizo ningún esfuerzo				Si eso todo esfuerzo

1	2	3	4	5
Ningun				Un gigante esfuerzo

Por favor haga un círculo en la palabra de la izquierda que significa 'la menos cantidad de esfuerzo' para usted.

Por favor haga un círculo en la palabra que significa 'la mayor cantidad de esfuerzo' para usted.

**Escogería usted diferentes palabras? Cuales serian?**

Finalmente nos gustaría tener alguna información de usted.

*** Su información es anónima, y será guardada de una manera segura. ***

Cual es su genero?
[ ] Femenino
[ ] Masculino

Cuantos años tiene? ________

Cual es el grado de educación usted completo, o el título más alto que usted recibió?
[ ] No se terminó el colegio, o menos de un año.
[ ] Guardería, jardín, y primaria (grados 1-8)
[ ] Colegio secundario (grados 9-12, sin terminar)
[ ] Colegio secundario completo (o equivalente)
[ ] Algo de Universidad (1-4 años, sin titulo)
[ ] Título técnico (incluyendo títulos ocupacionales o títulos académicos)
[ ] Título de bachiller (BA, BS, AB, etc)
[ ] Título de máster (MA, MS, MENG, MSW, etc)
[ ] Título de colegio profesional (MD, DDC, JD, etc)
[ ] Título de doctorado (PhD, EdD, etc)
[ ] Otros. Por favor especifique:

Cual es su ocupación? ________________________________

Cual es su etnicidad?
[ ] Hispano o Latino
[ ] No Hispano o Latino

Cual es su raza?
Por favor marque todo lo que aplique.
[ ]Indio americano o nativo de alaska.
[ ] Asiatico.
[ ] Negro o Africano americano.
[ ] Nativo de Hawaii u otro isleño del Pacifico.
[ ] Blanco.
[ ] Otro. Por favor especifique ________________________________

Que idioma (s) usted habla en casa?
Por favor marque todo lo que aplique.
[ ] Ingles.
[ ] Otro. Por favor especifique. ________________________________

Interview Topic Guide

At the beginning of the audio recording, "Escena__ Entrevista__. Nosotros estamos grabando sus repuestas y usted sabe y esta de acuerdo a ser grabado," a lo cual el participante puede responder "si" si el participante da su consentimiento.

To be read to interviewee:

Bueno, me gustaría empezar leyendo el párrafo que esta delante de usted. Queremos hacer de cuenta que usted es el siguiente paciente:

Usted ha ido a una clínica por un problema de salud para saber que se puede hacer. Usted sabe que hay unos cuentos tratamientos diferentes disponibles pero quiere saber mas acerca de ellos. A su salida a usted se le pide que complete este cuestionario.. El cuestionario puede venir en una página de papel, en una pantalla táctil o en su teléfono. No se le pedirá que de su nombre.

Abajo están algunas preguntas que pueda que se le pregunte al paciente (1-3). Imagine que usted es el paciente.

For each question, one at a time:

Por favor lea pregunta # __. No trate de contestar la pregunta. Nosotros solo queremos averiguar cuan clara y entendible es la pregunta. Por favor siéntase libre de pensar en voz alta y/o escribir sus ideas. Nosotros podremos discutir sus ideas una vez que usted haya tenido algo de tiempo para pensar acerca de cada pregunta.

Give the interviewee the interview schedule. Read through this with the interviewee. Give them interviewee some time to think about the question

Probes:

General Probes: Puede usted explicar? For favor dígame mas. Que quiere decir al mencionar eso?

Item 1.

1. Fue la pregunta clara y entendible?
2. En sus palabras que piensa que la pregunta está haciendo?
3. Que significa la frase "cuanto esfuerzo" para usted?
4. Que significa la frase" ayudarle a entender" para usted?
5. Que significa la frase "problemas de salud" para usted?
6. Usted prefiere "problemas de salud" o "estado de salud"?
7. Hay algo acerca de la pregunta que usted encuentra confuso o mal expresado?

Item 2.

1. En sus palabras que piensa usted que la pregunta está haciendo?
2. Que significa la frase "escuchar a las cosas que más le importan a usted" para usted?
3. Hay algo acerca de la pregunta que usted encuentra confuso o mal expresado?

Item 3.

1. En sus palabras que piensa que la pregunta está haciendo?
2. Que significa la frase " incluir lo que a usted le importa" para usted?
3. Que significa la frase "en la elección qué hacer a continuación"?
4. Hay algo acerca de la pregunta que usted encuentra confuso o mal expresado?

Response Scale:

Ask the interviewee to read the four response scales.

Por favor haga un círculo a la palabra a la izquierda que significa 'la menos cantidad de esfuerzo' para usted.
Por favor haga un círculo a la palabra a la derecha que significa 'la mayor cantidad de esfuerzo' para usted.
Usted Escogería palabras diferentes? Cuales serian?

Ask the patient to complete the demographics information sheet.

At the end of the audio recording, please state: "Gracias por su participación en esta entrevista. Por favor confirme que usted ha recibido la tarjeta de regalo de $10.00 de Market Basket.," to which the participant can reply "si" to confirm.
