# Supplementary material for: Developing and Pilot Testing a Spanish Translation of CollaboRATE for Use in the United States
Source: PLoS One. 2016 Dec 21;11(12):e0168538. doi: 10.1371/journal.pone.0168538 (PMC5176178; doi:10.1371/journal.pone.0168538)
Supplement: S2 File — (PDF) [file pone.0168538.s002.pdf]

En este cita, crees que…
(Por favor marque una caja por pregunta.)

	No se hizo 0	1	2	3	4	5	6	7	8	Se hizo lo mejor posible 9
¿Cuánto cree que se hizo para ayudarle a entender sus problemas de salud?	☐	☐	☐	☐	☐	☐	☐	☐	☐	☐
¿Cuánto cree que se hizo para escucharle cuando usted comunico lo que mas le importa acerca de sus problemas de salud?	☐	☐	☐	☐	☐	☐	☐	☐	☐	☐
¿Cuánto cree que se hizo para incluir lo que mas le importa a usted cuando se escogió el siguiente paso?	☐	☐	☐	☐	☐	☐	☐	☐	☐	☐

Sexo: ☐ Hombre ☐ Dama

Edad: ☐

¿Tenía un intérprete médico le ayuda durante este cita? ☐ Sí ☐ No

---

Office use only:

Pod A	Pod B	Pod C	Pod D	
☐ LB	☐ EA	☐ WA	☐ MC	☐ Other
☐ LC	☐ VL	☐ HC	☐ CF	
☐ JM	☐ SP	☐ LP	☐ JZ	
☐ PP	☐ DS	☐ LB	☐ EM	
		☐ MM		

00A
