# Supplementary material for: Developing and Pilot Testing a Spanish Translation of CollaboRATE for Use in the United States
Source: PLoS One. 2016 Dec 21;11(12):e0168538. doi: 10.1371/journal.pone.0168538 (PMC5176178; doi:10.1371/journal.pone.0168538)
Supplement: S1 Table — (PDF) [file pone.0168538.s003.pdf]

CollaboRATE question 1 response	English questionnaire respondents (n)	Spanish questionnaire respondents (n)
**0**	1	0
**1**	0	0
**2**	0	1
**3**	0	1
**4**	3	1
**5**	6	4
**6**	3	4
**7**	20	15
**8**	38	31
**9**	553	549
