# Supplementary material for: Developing and Pilot Testing a Spanish Translation of CollaboRATE for Use in the United States
Source: PLoS One. 2016 Dec 21;11(12):e0168538. doi: 10.1371/journal.pone.0168538 (PMC5176178; doi:10.1371/journal.pone.0168538)
Supplement: S2 Table — (PDF) [file pone.0168538.s004.pdf]

**CollaboRATE question 2 response**	**English questionnaire respondents (n)**	**Spanish questionnaire respondents (n)**
**0**	2	0
**1**	0	0
**2**	0	1
**3**	1	1
**4**	2	1
**5**	4	6
**6**	2	4
**7**	14	12
**8**	35	33
**9**	564	548
