# Supplementary material for: Developing and Pilot Testing a Spanish Translation of CollaboRATE for Use in the United States
Source: PLoS One. 2016 Dec 21;11(12):e0168538. doi: 10.1371/journal.pone.0168538 (PMC5176178; doi:10.1371/journal.pone.0168538)
Supplement: S3 Table — (PDF) [file pone.0168538.s005.pdf]

CollaboRATE question 3 response	English questionnaire respondents (n)	Spanish questionnaire respondents (n)
**0**	1	0
**1**	0	0
**2**	0	1
**3**	0	0
**4**	5	3
**5**	4	4
**6**	4	2
**7**	16	12
**8**	30	38
**9**	564	546
