# Supplementary material for: Developing and Pilot Testing a Spanish Translation of CollaboRATE for Use in the United States
Source: PLoS One. 2016 Dec 21;11(12):e0168538. doi: 10.1371/journal.pone.0168538 (PMC5176178; doi:10.1371/journal.pone.0168538)
Supplement: S4 Table — (PDF) [file pone.0168538.s006.pdf]

Respondent age	English questionnaire respondents (n)	Spanish questionnaire respondents (n)
**1**	0	1
**4**	0	1
**5**	0	1
**9**	0	1
**10**	0	1
**11**	0	2
**18**	2	1
**19**	7	0
**20**	7	5
**21**	14	7
**22**	12	5
**23**	15	8
**24**	8	6
**25**	10	5
**26**	8	10
**27**	14	10
**28**	12	9
**29**	10	9
**30**	3	7
**31**	9	14
**32**	4	12
**33**	11	12
**34**	15	16
**35**	10	10
**36**	6	16
**37**	11	14
**38**	10	20
**39**	10	11
**40**	6	13
**41**	7	10
**42**	3	11
**43**	4	11
**44**	6	9
**45**	11	16
**46**	4	18
**47**	11	20
**48**	11	12
**49**	8	21
**50**	13	14
**51**	9	9

**52**	10	10
**53**	15	12
**54**	18	9
**55**	14	13
**56**	11	7
**57**	12	12
**58**	8	9
**59**	14	5
**60**	16	9
**61**	16	5
**62**	14	4
**63**	7	8
**64**	9	5
**65**	8	5
**66**	6	15
**67**	8	5
**68**	15	7
**69**	8	5
**70**	11	9
**71**	5	2
**72**	10	9
**73**	8	2
**74**	7	4
**75**	6	5
**76**	6	3
**77**	4	5
**78**	6	6
**79**	5	3
**80**	6	5
**81**	9	4
**82**	2	3
**83**	4	1
**84**	2	3
**85**	4	0
**86**	3	4
**87**	3	2
**88**	4	2
**89**	3	0
**90**	2	0
**91**	0	2
**92**	0	0

**93**	1	0
**94**	1	0
**95**	1	0
**96**	0	1
**97**	0	0
**98**	0	1
**99**	0	1
